# Enhancing the antibacterial activities of sow milk via site-specific knock-in of a lactoferrin gene in pigs using CRISPR/Cas9 technology

**DOI:** 10.1186/s13578-020-00496-y

**Published:** 2020-11-19

**Authors:** Xiaosong Han, Yang Gao, Guanglei Li, Youcai Xiong, Changzhi Zhao, Jinxue Ruan, Yunlong Ma, Xinyun Li, Changchun Li, Shuhong Zhao, Shengsong Xie

**Affiliations:** 1 Key Laboratory of Agricultural Animal Genetics, Breeding and Reproduction of Ministry of Education & Key Lab of Swine Genetics and Breeding of Ministry of Agriculture and Rural Affairs, Huazhong Agricultural University, Wuhan, 430070 People’s Republic of China; 2 The Cooperative Innovation Center for Sustainable Pig Production, Huazhong Agricultural University, Wuhan, 430070 People’s Republic of China

**Keywords:** Pig, Lactoferrin, Colostrum, CRISPR/Cas9, Homologous recombination

## Abstract

Colostrum quality is a vital factor in mortality and growth performance for piglets. Lactoferrin is an immuno-active milk protein that contributes to the formation of a protective layer above intestinal mucosa, possesses the antibacterial and antiviral activities that are favorable for piglet development. However, there is a notable reduction in lactoferrin in sow milk during lactation after the first few days, which causes many piglets to fail to ingest enough colostrum thereby leading to an increase in piglet mortality. In this study, we successfully constructed genome-edited Large-White pigs with marker-free site-specific knock-in of *lactoferrin* gene in the 3′-end of *Casein alpha*-*s1* via CRISPR/Cas9 mediated homologous recombination. Thus, the lactoferrin protein can be expressed in the mammary gland in the control of *Casein alpha*-*s1* promoter. As expected, the lactoferrin protein in genetically modified pigs sustained high expression in both colostrum and milk when compared with wild-type pigs. Moreover, the bacterial plate assay indicated that the milk from genetically modified pigs showed bacteriostatic effects when compared with control pigs. Taken together, our study demonstrated that the milk from genetically modified pigs had antibacterial activity which may reduce the costs of veterinary drug and improve the surviving rate of piglets, which is promising for pig breeding.

Dear Editor,

Due to the lack of a fully developed immune system, newborn piglets are susceptible to pathogenic bacteria, thus causing billions of dollars in annual global losses. Sow milk, especially colostrum, containing abundant immune active compounds, plays a key role in piglet thermoregulation, acquisition of passive immunity, and intestinal development [1]. Colostrum quality is a vital factor in mortality and growth performance for piglets. Lactoferrin (LF) is an immune-active milk protein that contributes to the formation of a protective layer above intestinal mucosa, possesses antibacterial and antiviral properties [2], therefore, piglets suffer less from intestinal inflammation or diarrhea. However, the secretion volume for porcine lactoferrin (pLF) decreases in sow milk along with the lactation. Previous studies have shown that artificial supplementation of the piglet diet with LF can enhance piglet growth [3]. We assume that overexpression of the *LF* gene in sow milk might help to improve piglet survival rate. To demonstrate this hypothesis, we established gene-edited pigs with the targeted insertion of *LF* using CRISPR/Cas9-mediated knock-in system.

Overexpressing *LF* using transgenesis methods have been reported in many species, such as dairy cattle, goat, and mouse. In 2015, Cui et al. reported the production of transgenic pigs overexpressing human LF which brought LF content to reach 6.5 g/L [4]. While, most of the researches used random insertion of the *LF* gene, which arose safety concerns for the animals. In recent years, many kinds of genetically modified animals using CRISPR/Cas9 technology have been generated to improve animal traits [5]. Inspired by these studies, we attempted to generate genetically modified pigs that could produce LF at a high level throughout the entire lactation period. To minimize the effect of the inserted gene, we intended to insert the *pLF* gene before the stop codon site of the *Casein alpha*-*s1* gene (CSN1S1) linked by a self-cleavage peptide, P2A sequence (Fig. 1a). CSN1S1 is expressed specifically in the mammary gland and sustains high expression during lactation [6]. Thus, the promoter of *CSN1S1* was employed to drive the expression of *pLF*, which may minimize the effect in other tissues.

**Fig. 1:**
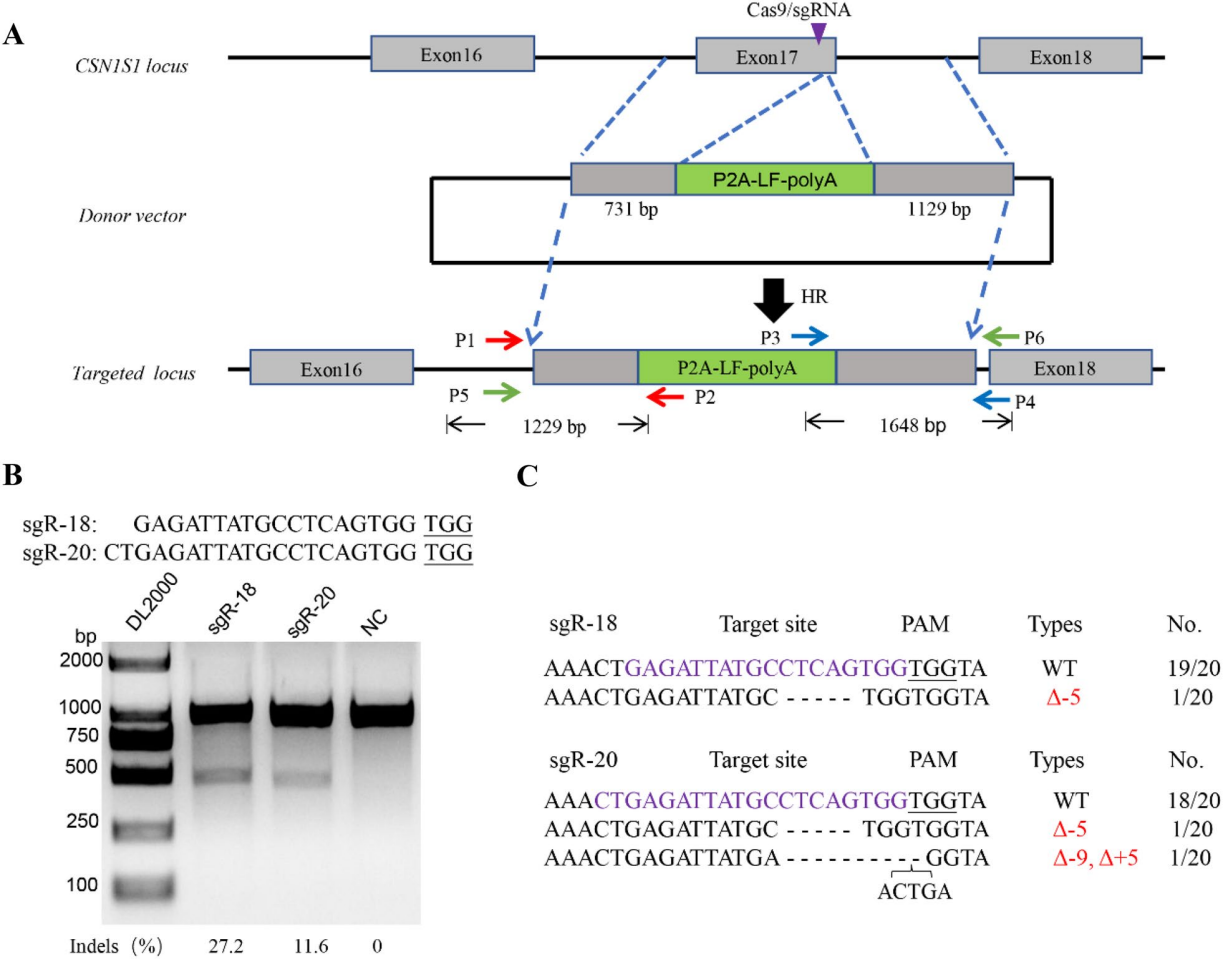
Construction of CRISPR plasmids and activity assessment. **a** Schematic representation of the design of the CSN1S1-targeting vector. Exons of *CSN1S1* and homologous arms are shown as gray boxes, and the purple triangle in Exon 17 represents the sgRNA targeting site. The *pLF* gene flanked by P2A and polyA in green is located in the middle of the targeting vector. Three pairs of primers were designed for genotyping. P1/P2 and P3/P4 are designed for genotyping the 5′ and 3′ junctions in pLF-KI colonies, respectively. P5/P6 are designed for detecting the donor vector region within the genetically modified pigs. LF, lactoferrin; HR, Homologous Recombination; sgRNA, single-guide RNA. **b** Assessment of the activity of 18- and 20-nt sgRNAs targeted to the same genomic loci via the T7ENI cleavage assay in vitro. The upper sequences indicate the 18- and 20-nt sgRNAs, respectively, with the protospacer adjacent motif (PAM) sequence underlined. NC, negative control; M, DL2000 Marker. **c** Verification of activity of different length sgRNAs by Sanger sequencing. Characters in purple indicate the sgRNA sequences; The PAM sequences are underlined; Δ, deletion; +, insertion; WT, wild-type; No., Numbers of Sanger sequencing results

## Construction of CRISPR plasmids and assessment of its activity

To verify this strategy, we constructed a homologous recombination donor vector targeting the *CSN1S1* locus in the pig genome. The single-guide RNA (sgRNA) closest to the stop codon was selected for construction of sgRNA-expressing vector. Previous studies showed that the length of the sgRNA may affect genome editing activity [7, 8]. To optimize the efficiency of the sgRNA, one 18-nt sgRNA and another 20-nt sgRNA were synthesized and assembled. A T7 endonuclease I (T7ENI) cleavage assay was used to evaluate the targeting efficiency for the two sgRNAs (Fig. 1b). Interestingly, when the length of sgRNA was decreased to 18-nt, the cleavage efficiency was more than 2-fold than that of a 20-nt sgRNA. Then, Sanger sequencing was used to identify the mutation (Fig. 1c). Therefore, we chose the 18-nt sgRNA for subsequent experiments. To achieve *pLF* knock-in at the 3′-end of the *CSN1S1* locus, a homologous recombination donor vector was constructed (Fig. 1a). We chose the modified PCI vector with a deleted promoter as the backbone. The left homologous arm was approximately 731 bp and the right homologous arm was approximately 1129 bp. We used P2A as the linker between the *pLF* coding sequence (CDS) and the left homologous arm.

## Generation of site-specific lactoferrin knock-in pigs using CRISPR/Cas9 technology

**Fig. 2:**
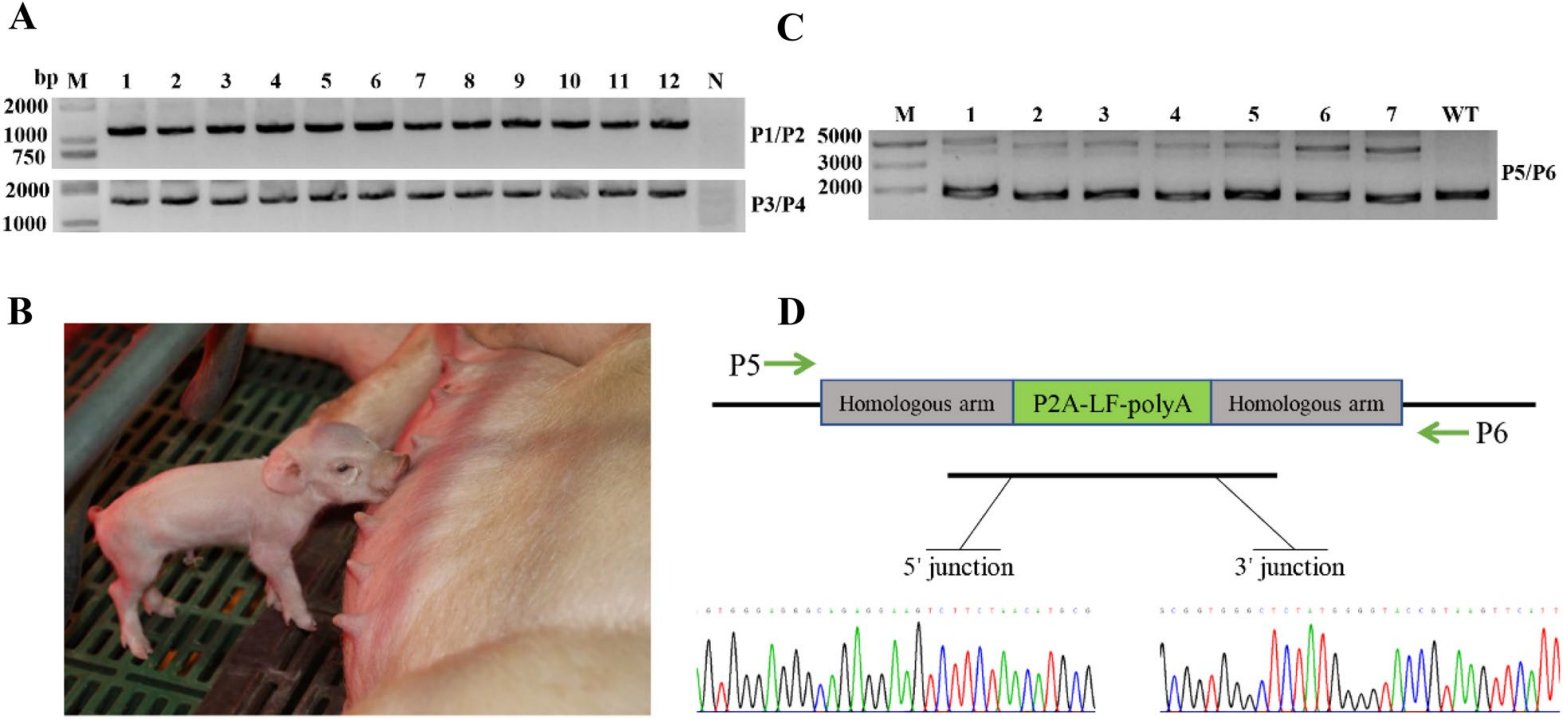
Generation of site-specific lactoferrin knock-in pigs using CRISPR/Cas9 technology. **a** Detection of site-specific insertion of selected clones using PCR genotyping. Lanes 1–12 represent 12 different clones, lanes 2, 3, 7, 9, 10 represent clone 25#, 27#, 68#, 75# and 81#, respectively; lane N represents the negative control wild-type cell line. **b** A genetically modified piglet at day 3 postpartum. **c** Detection of site-specific insertion of donor DNA in the genetically modified pigs using a PCR genotyping assay. Lanes 1–7 represent 7 individuals, and lane WT represents one negative control wild-type pig. WT, wild-type. **d** Nucleotide sequence analysis of junctions between endogenous and exogenous DNA corresponding to homologous recombination (HR) events. P5/P6 primers were used to amplify the specific region for the left- and right-hand junctions. Primary nucleotide sequence data corresponding to transition regions between the homologous arms of the targeting vector and the internal transgene DNA. LF, lactoferrin

Porcine fetal fibroblast (PFF) cells isolated from 35-day-old embryos of Large-White pigs were electroporated with circular plasmids of CSNIS1-sgRNA, Cas9, and donor vector. We used G418 selection (400 μg/mL for 10 days) to identify the positive cell colonies, and their genotype was identified using PCR and Sanger sequencing. Among the 109 surviving cell colonies, twelve of them (12/109, 11%) were identified as positive for *pLF* insertion (Fig. 2a and Additional file 1: Figure S3A). Next, the verified colonies were used as donor cells for nuclear transfer. A total of 1750 cloned embryos were successfully reconstructed in vitro, and 39% of which were developed into blastocysts (Additional file 1: Figure S1). Subsequently, these blastocysts were transferred into 7 surrogate mothers (Additional file 1: Table S2). Fortunately, five surrogates were confirmed pregnant through ultrasound detection 35 days post embryo transfer. Finally, all of these surrogates developed to term and gave birth to 25 genetically modified piglets (Fig. 2b). Genomic DNA was extracted from the ear tissues of these cloned piglets 3 days after birth, and their genotype was identified by PCR (Fig. 2c and Additional file 1: Figure S3B), the results showed that all of the piglets were heterozygous. The sequencing results further confirmed the desired precise knock-in location in the genetically modified piglets (Fig. 2d). Furthermore, to detect whether there were off-target effects in the cloned piglets, potential off-targets (OTS) were predicted by CRISPR-offinder [9]. A total of 20 putative OTS with less than 4 mismatches were considered and were examined via T7ENI cleavage assay, some of the OTS were furtherly verified by Sanger sequencing (Additional file 1: Figure S2). No mutations were found at the potential off-target sites in all of the genetically modified piglets. These results indicated that seamless site-specific modification for the *pLF* gene in pig had been successfully achieved. Furthermore, this results in modified pigs with minimal safety concerns for further pig breeding in agriculture.

## Detection of the antibacterial activity of milk from pLF-KI pigs in vitro

**Fig. 3:**
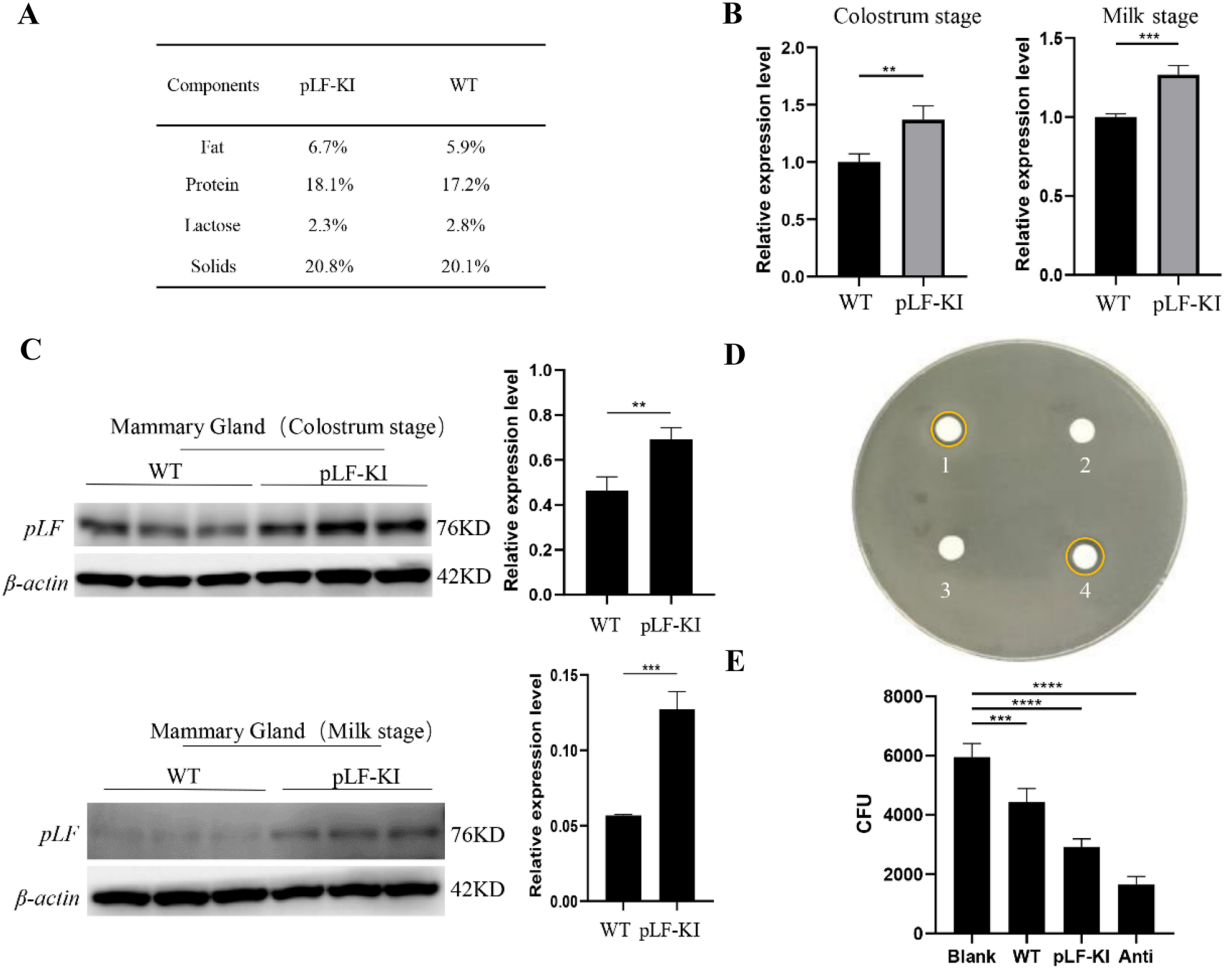
Detection of the antibacterial activity of milk from pLF-KI pigs in vitro. **a** Raw components of genetically modified colostrum compared with wild-type colostrum. **b** Detection of the expression of *LF* in colostrum stage and milk stage in pLF-KI pigs by qRT-PCR assay. **c** Western blot analysis for the expression and accumulation of LF protein between pLF-KI pigs and wild-type pigs. KD, Kilodalton. **d** Bacterial plate assay for *Escherichia coli* (*E. coli*) survival in response to colostrum treatment. Depletion zones developed on a lawn of *E. coli* 12 h after sample application: (1) 10 μL milk from genetically modified pig, (2) 10 μL milk from wild-type pig, (3) 10 μL sterile water and (4) 10 μL antibiotic. **e** Plate dilution colony counting assay to test the antibacterial activity of colostrum. pLF-KI, porcine lactoferrin knock-in; WT, wild-type; Anti, Antibiotic; CFU, colony-forming unit; Blank: sterile water; the cycles in orange indicate the transparent zones. ^**^P < 0.01, ^***^P < 0.001, ^****^P < 0.0001; *P* values were determined by two-sided Student’s *t* test

To test whether *pLF* gene expression was up-regulated in the mammary gland tissue during the lactation period, three sexually mature genetically modified sows were artificially inseminated at 7-8 months old. One of the sows was pregnant and gave birth to 7 piglets. Three of them were demonstrated as pLF-KI pigs. Firstly, we analyzed the quality of colostrum collected from genetically modified and wild-type pigs. The results showed that there was no significant difference in the percentage of fat, protein, lactose, and solids in the colostrum of pLF-KI and wild-type pigs (Fig. 3a). Mammary gland tissue was collected on day 1 (colostrum stage) and day 12 (milk stage) after parturition. To determine the relative transcriptional expression levels of the *pLF* gene in pLF-KI pigs, qRT-PCR assays were performed. The results showed that the *LF* gene expression was significantly higher during the pLF-KI sow lactation period (Fig. 3b). In addition, Western blot assays of mammary tissue also showed that LF protein accumulated to high abundance in both the colostrum and milk of pLF-KI pigs compared to that of wild-type pigs (Fig. 3c). Additionally, we performed antibacterial experiments to determine the function of the colostrum from the genetically modified pig. Filter paper containing colostrum was plated on the agar plates containing *Escherichia coli* (*E. coli*) and the antibacterial activity was estimated by the presence of transparent zones appearing around the filter paper after a 24 h incubation at 37 °C (Fig. 3d), we found that the pLF-KI milk produced a transparent zone, while no transparent zone was found for the wild-type milk. Subsequently, the antibacterial activity was also verified by plate dilution colony counting (Fig. 3e). These results indicated that the milk from genetically modified pigs had bacteriostatic effects when compared to the wild-type control.

## Conclusion

In summary, we successfully generated seamlessly-engineered pigs by CRISPR/Cas9-mediated homologous recombination, and we knocked in the *pLF* gene at the 3′-end of endogenous *CSNIS1* locus in the pig genome for the first time. Thus, the *pLF* gene can be overexpressed specifically in the mammary gland during the lactation period under the control of the *CSN1S1* promoter. Also, this study demonstrated that the milk from genetically modified pig possessed antibacterial activity without significant changes to its nutritional composition, which may reduce the costs associated with veterinary drug treatment and improve the mortality rate of piglets. Our work demonstrates a promising strategy for pig breeding and trait improvement efforts and provides hope that this strategy can be adopted in other species in the future.

## **Supplementary information**

**Additional file 1**: **Table S1.** Primer pairs used in this study. **Table S2.** Summary of embryo transfer for the generation of genetically modified pigs. **Figure S1**. The porcine reconstructed embryos were cultured in vitro. **Figure S2.** Detection of predicted off-target sites mutation by the T7ENI cleavage assay. **Figure S3**. Genotyping of cell clones and genetically modified pigs by PCR.

## Data Availability

All relevant data are within this paper.

## Abbreviations

CRISPR: Clustered Regularly Interspaced Short Palindromic Repeats
sgRNA: single-guide RNA
LF: lactoferrin
CDS: coding sequences
PAM: protospacer adjacent motif
KI: knock-in
CSN1S1: casein alpha-s1
HR: homologous recombination
WT: wild-type
PFF: Porcine fetal fibroblast
PCR: polymerase chain reaction
OTS: potential off-targets
SCNT: somatic cell nuclear transfer
CFU: colony-forming unit
qRT-PCR: Real-time reverse transcription PCR
cDNA: complementary DNA
