# Supplementary material for: Enhancing the antibacterial activities of sow milk via site-specific knock-in of a lactoferrin gene in pigs using CRISPR/Cas9 technology
Source: Cell Biosci. 2020 Nov 19;10:133. doi: 10.1186/s13578-020-00496-y (PMC7678085; doi:10.1186/s13578-020-00496-y)
Supplement: Supplementary file 1 — Additional file 1: Table S1. Primer pairs used in this study. Table S2. Summary of embryo transfer for the generation of genetically modified pigs. Figure S1. The porcine reconstructed embryos were cultured in vitro. Figure S2. Detection of predicted off-target sites mutation by the T7ENI cleavage assay. Figure S3. Genotyping of cell clones and genetically modified pigs by PCR. [file 13578_2020_496_MOESM1_ESM.doc]

**Additional file 1**

**Materials and Methods**

**Ethics statement.** In this study, all pigs were raised under conventional housing conditions at the Animal Research Center of Huazhong Agricultural University. All experiments were conducted according to the guidelines for the care and use of animals by the Huazhong Agricultural University Institutional Animal Care and Use Committee. We tried every effort to minimize animal pain, suffering, and distress and to reduce the number of animals used.

**Plasmid construction.** The vector expressing humanized Cas9 (hCas9) was purchased from Addgene (#41815). The sgRNA-expressing vector containing the U6 promoter and the sgRNA scaffold (pGL3-U6-sgRNA-PGK-puromycin) was a gift from Prof. Xingxu Huang (Addgene, #51133). The PCI vector was a gift from Prof. Hong Wei (Genbank: U47119). The sgRNA-expressing vector was digested via restriction enzyme (*Bbs*I). A pair of complementary oligos for the target site were synthesized, annealed, and ligated into the linearized sgRNA-expressing vector. To construct the donor vector, the PCI vector was digested with *Bgl*II and *Nhe*I, and the linearized vector was gel-purified. Then, the upstream homologous arm, self-cleaving P2A peptide, *LF* gene CDS cloned from pig mammary gland tissue cDNA, the polyA terminal sequence, and the downstream homologous arm were tandemly ligated one by one into the linearized PCI vector to generate the donor vector. All constructs were verified by Sanger sequencing.

**Cell culture and plasmids transfection.** All the cells used in this study were maintained at 37℃ and 5% CO_2_ in a humidiﬁed incubator. Porcine fetal fibroblast (PFF) cells were isolated from 35-day-old embryos of large-white pigs according to the previous study [1]. The PFF cells were maintained in DMEM media supplemented with 15% Fetal bovine serum (FBS), 1% penicillin/streptomycin, 1% Non-essential Amino Acids, 2 mM GlutaMax, and 1mM sodium pyruvate, and were culture in a humidified incubator. PFFs were seeded onto 10 cm dishes one day before transfection, and when the cells were 90% confluent, approximately 1×10^6^ cells were electroporated via the Neon transfection system (Life Technology) at 1350 V for 30 ms with 1 pulse in 100 μL of Buffer R containing 8 μg Cas9 vector, 4 μg CSN1S1-sgRNA vector, and 5 μg donor vector. After electroporation, cells were plated onto 10 cm dishes, and 48 h later, 400 μg/mL G418 (Merck) was added in the culture medium. The cell colonies appeared after about 10 days. Then the cell colonies were isolated by cloning cylinders (Corning) and seeded into 24-well plates. Next, when the cells grew up, half of the cells were collected for genotyping and the rest of the cells were plated into 12-well plates. After sequencing, the positive colonies were frozen for somatic cell nuclear transfer (SCNT). All primers used in this study are list in Table S1.

**Genotyping of cell colonies.** Genomic DNA was extracted from cell colonies after lysis in 10 μL NP40 solution (0.45% NP40 and 0.6% Proteinase K) at 56°C for 60 min and 95°C for 10 min. The primers P1/P2 and P3/P4 were designed and used to amplify the 5’-end and 3’-end junctions, respectively. The PCR products were gel-purified and sequenced. The positive colonies were frozen for SCNT.

**T7EN**I **cleavage assay and Sanger sequencing.** To optimize the activity of sgRNA, two sgRNAs with lengths of 18 nts and 20 nts were designed by CRISPR-offinder [2], and they were cloned into the PX330 (Addgene, #42230) vector. After transfection, cells were incubated for 48 h. Then, the genomic DNA of the cells was extracted with the TIANamp Genomic DNA kit (TIANGEN, China). The primers P7/P8 were used to amplify the targeted region to generate a mixture of wild-type and mutant amplicons, and then, the PCR products were gel-purified. Then, the PCR products were denatured and annealed in NEB buffer 2 by using a thermocycler under the following conditions: 95℃ for 10 min, 95℃ to 85℃ ramping at −2 ℃/s, 85℃ to 25℃ at −0.25℃/s, and held at 15℃ for 2 min. Hybridized PCR products were digested with T7ENI at 37℃ for 10 min and were then analyzed on a 2% agarose gel.

**Nuclear transfer and embryo transfer.** Pig ovaries were collected from a local slaughterhouse and transported to the laboratory within 2 h in 0.9% saline maintained at 37℃. The protocols used for porcine oocyte collection, *in vitro* maturation, and SCNT were described in a previous work [3]. The surrogate mothers were examined 35 days post-embryo transfer via ultrasound.

**Western blot analysis.** Mammary gland tissues were collected on day 1 and day 12 after parturition via a breast biopsy probe, and the tissues were dissected, frozen immediately in liquid nitrogen, and stored at -80℃. An equal amount of the total protein from each sample was separated 12% sodium dodecyl-sulphate polyacrylamide gel electrophoresis (SDS-PAGE) and transferred to a polyvinylidene diﬂuoride membrane (Millipore, Germany). Then, membranes were blocked with 5% non-fat milk and incubated with β-actin (Santa Cruz, USA) and lactoferrin (Bioss, China) antibody at dilution of 1:1000. After washing with TBST, HRP-conjugated anti-Rabbit IgG was used as the secondary antibody. Digital signal of chemiluminescent Western blotting was acquired by Bio-Rad GelDoc XR and ChemiDoc XRS system, and analysis was conducted by the Quantity One program (Bio-Rad, USA).

**Bacterial plate assay.** The bacterial plate assay used for determine the antibacterial activity of milk was referred to previous described method [4]. Briefly, milk from pLF-KI pigs and wild-type pigs were harvested on day 1 and day 12 after parturition. LB agar-plate containing *Escherichia coli* were used to examine the antibacterial activity of the milk. Six-mm filter papers were plate on the surface of the plate and 10 μL milk from genetically modified pig, 10 μL milk from wild-type pig, 10 μL sterile water and 10 μL antibiotic (Ampicillin, 100mg/mL) were pipetted onto the filter paper. After a 24 h incubation at 37℃, bacteriostatic activity was assessed from the area of growth inhibition surrounding the discs.

**Real-time reverse transcription PCR (qRT-PCR) analysis.** Total RNA from mammary gland tissue was extracted with TransZol Up (Transgen, China), and measured by NanoDrop 2000 spectrophotometer (Thermo Scientific, USA) for assessing RNA quantity and quality. cDNAs were synthesized using the PrimeScript^TM^ RT reagent Kit with gDNA Eraser (TaRaKa, Japan). Then, the cDNA products used as templates for qRT-PCR. The qPCR reactions were prepared with RealUniversal SYBR Green Premix (TIANGEN, China) following the manufacturer’s instructions. Briefly, PCR mixtures (10 μL) contained 5 μL RealUniversal Premix, 0.3μL forward primer (0.3 μM), 0.3 μL reverse primer (0.3 μM) and 1 μL cDNA template. The results were monitored using a CFX96 Real-Time PCR Detection System (Bio-Rad, USA) programmed for one cycle of 15 min at 95℃，followed by 39 cycles of 10 s at 95℃, 30 s at 60℃. The glyceraldehyde-3-phosphate dehydrogenase (GAPDH) gene was used as a normalization control. Relative expression levels were calculated using the 2^-ΔΔCt^ method.

**References**

1. Yao, J., et al., Efficient bi-allelic gene knockout and site-specific knock-in mediated by TALENs in pigs. Sci Rep, 2014. **4**: p. 6926.

2. Zhao, C.Z., et al., CRISPR-offinder: a CRISPR guide RNA design and off-target searching tool for user-defined protospacer adjacent motif. International Journal of Biological Sciences, 2017. **13**(12): p. 1470-1478.

3. Zhou, X., et al., Generation of CRISPR/Cas9-mediated gene-targeted pigs via somatic cell nuclear transfer. Cell Mol Life Sci, 2015. **72**(6): p. 1175-84.

4. Cui, D., et al., Generation of bi-transgenic pigs overexpressing human lactoferrin and lysozyme in milk. Transgenic Research, 2015. **24**(2): p. 365-373.

Table S1. Primer pairs used in this study

| **Primers** | **Sequences (5’-3’)** | **Annealing temperature** |
| --- | --- | --- |
| PX330-CSN1S1-sgR-18-S | caccGAGATTATGCCTCAGTGG | \ |
| PX330-CSN1S1-sgR-18-A | aaacCCACTGAGGCATAATCTC |
| PX330-CSN1S1-sgR-20-S | caccGCTGAGATTATGCCTCAGTGG | \ |
| PX330-CSN1S1-sgR-20-A | aaacCCACTGAGGCATAATCTCAGC |
| P1 | AAGGGTACACTAACCTCAACTTT | 64℃ |
| P2 | CAGCCAGACACAGTCCAAGT |
| P3 | GCCCAACTCCAAGGCAAAAC | 64℃ |
| P4 | ACAGAGAAAATATGGCCAAGGAA |
| P5 | ACCACAGCAAAACTGGAAATG | 62℃ |
| P6 | TGGCCAAGGAATTCAAAGAAAAA |
| P7 | TGGGCTCTGGTAACTGGACT | 64℃ |
| P8 | AGGTCAGGATTTGGATTTGGGA |
| CSN1S1-OT01-735-F | GCCAGAAATCAGAACGGCAC | 60℃ |
| CSN1S1-OT01-735-R | GAGCAGCTACCTATGCCAGC |
| CSN1S1-OT02-752-F | TGGCTGGGAAGAAGAGTTTGA | 62℃ |
| CSN1S1-OT02-752-R | GCTTGCCCAGAGGATTAGGT |
| CSN1S1-OT03-627-F | TTTCGGGTTTGAGGGAGGTT | 62℃ |
| CSN1S1-OT03-627-R | AGAATTAGGCCCCCTCCCAT |
| CSN1S1-OT04-652-F | ATGCCTTTTGATGCCCAAGTG | 60℃ |
| CSN1S1-OT04-652-R | GTGTCCAGTATTCCGTTGGTCT |
| CSN1S1-OT05-644-F | GTCTCTGCCTGACGGTGTTG | 62℃ |
| CSN1S1-OT05-644-R | ACAACAGACGTTCCTGCCTT |
| CSN1S1-OT06-641-F | AGTTGGTAATCGCTGAGGCAG | 62℃ |
| CSN1S1-OT06-641-R | GTAGGAAGGACAGGCATCCA |
| CSN1S1-OT07-592-F | CATGGGGTTGCTTGTCCTTT | 62℃ |
| CSN1S1-OT07-592-R | ACCATCCATCAGGTGTGTCAA |
| CSN1S1-OT08-712-F | GGAAAGTGACATCCACTCTGAC | 60℃ |
| CSN1S1-OT08-712-R | TCCAGCTTTGACTACGGATGTG |
| CSN1S1-OT09-730-F | AGGTCAGAGCAGGCATCAC | 62℃ |
| CSN1S1-OT09-730-R | TTTAACTGAACCCCAGGCATGT |
| CSN1S1-OT10-703-F | TGGACGTTATTGCCCCTGTT | 64℃ |
| CSN1S1-OT10-703-R | AAGAAGCTCAGCCATTCCCG |
| CSN1S1-OT11-769-F | GCATCTTCTGCCCCCATGTT | 64℃ |
| CSN1S1-OT11-769-R | TGCAGCCACTTTGTTTGAGTG |
| CSN1S1-OT12-598-F | TGGGCTTAACAGGAACACCA | 64℃ |
| CSN1S1-OT12-598-R | AGATGGCCGCTTAGAAGGTTG |
| CSN1S1-OT13-694-F | GCCTGCCTACTTGTTGGAAT | 64℃ |
| CSN1S1-OT13-694-R | CTGTCAACACCAAGCTAACTAC |
| CSN1S1-OT14-713-F | GCTGAGGGAATCAGCAGTCA | 64℃ |
| CSN1S1-OT14-713-R | AGGCAATGGGCAGATAGTGG |
| CSN1S1-OT15-771-F | CAGGTCTTTGGTGTTCAGACG | 64℃ |
| CSN1S1-OT15-771-R | GCTGCTACATCCTGCCACTT |
| CSN1S1-OT16-634-F | TAAGTAGCGGAGACAAGGGCT | 62℃ |
| CSN1S1-OT16-634-R | CTTTGGGCTCTACAACTCGGT |
| CSN1S1-OT17-659-F | ACGACGGAGTTTCAAGAAGGA | 64℃ |
| CSN1S1-OT17-659-R | GCAAATGTTAGGGAACCTCGC |
| CSN1S1-OT18-653-F | GAAGGACCCCGAGACTCTTTT | 62℃ |
| CSN1S1-OT18-653-R | ATCTTGCTCGGCTCTGTTTTT |
| CSN1S1-OT19-806-F | AGGGCTTGCTTGAACACAGTA | 62℃ |
| CSN1S1-OT19-806-R | AATAAGGGACTTGGCAGTCGG |
| CSN1S1-OT20-863-F | TCGCCTCAACATTCATCTCAC | 60℃ |
| CSN1S1-OT20-863-R | GCTGAATGCTGAAAGGCTGATT |
| pLF-qPCR-166-F | CGGGACAGAAGAGAATCCCC | 60℃ |
| pLF-qPCR-166-R | GTCCAAGAACCGGCGAAGTA |
| GAPDH- qPCR-194-F | CGTCCCTGAGACACGATGGT | 60℃ |
| GAPDH- qPCR-194-R | GCCTTGACTGTGCCGTGGAAC |

**Table S2. Summary of embryo transfer for the generation of genetically modifi**ed pigs

| **Surrogate ID** | **Cell clone** | **No. of embryos transferred** | **Blastocysts**  **(%)** | **Gestation length (Days)** | **No. of pigs born** |
| --- | --- | --- | --- | --- | --- |
| P0565 | 27#, 81# | 250 | 39 | 115 | 1 |
| P4979 | 68#, 75# | 250 | 39 | 120 | 7 |
| P4982 | 25#, 27#, 81# | 250 | 39 | 118 | 5 |
| P4986 | 25#, 27#, 81# | 250 | 39 | 115 | 7 |
| P4988 | 68#,75# | 250 | 39 | 116 | 7 |
| P4992 | 68#, 75# | 250 | 39 | / | / |
| P4995 | 68#, 75# | 250 | 39 | / | / |

**
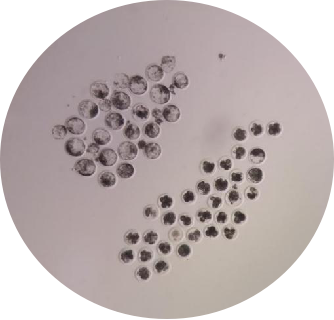
**

**Figure S1.** The porcine reconstructed embryos were cultured *in vitro*. Grade 1 blastocysts were collected in the upper left corner.


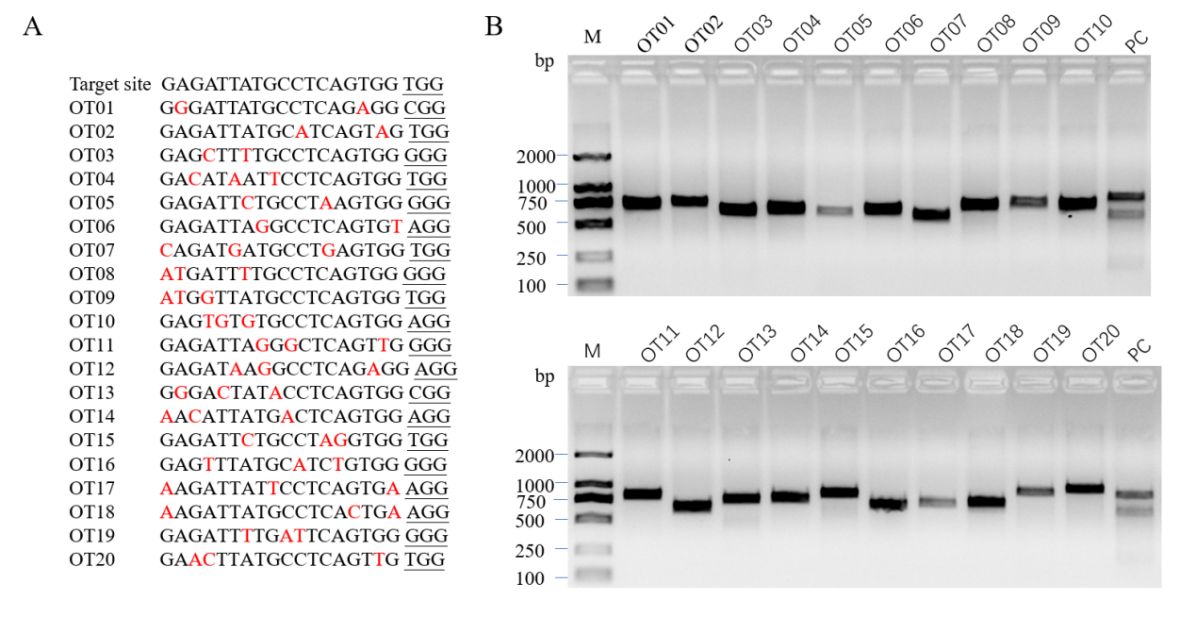


**Figure S2. Detection of predicted off-target sites mutation by the T7EN**I **cleavage assay. A**: Potential off-target site sequences. Characters in red indicate the mismatch with the sgRNA sequence. The underlined sequence indicates the PAM sequence. B: Detection of the predicted off-target sites by the T7ENI assay. POT, potential off-target site; PC, positive control; bp, base pair; M, DL2000 marker.


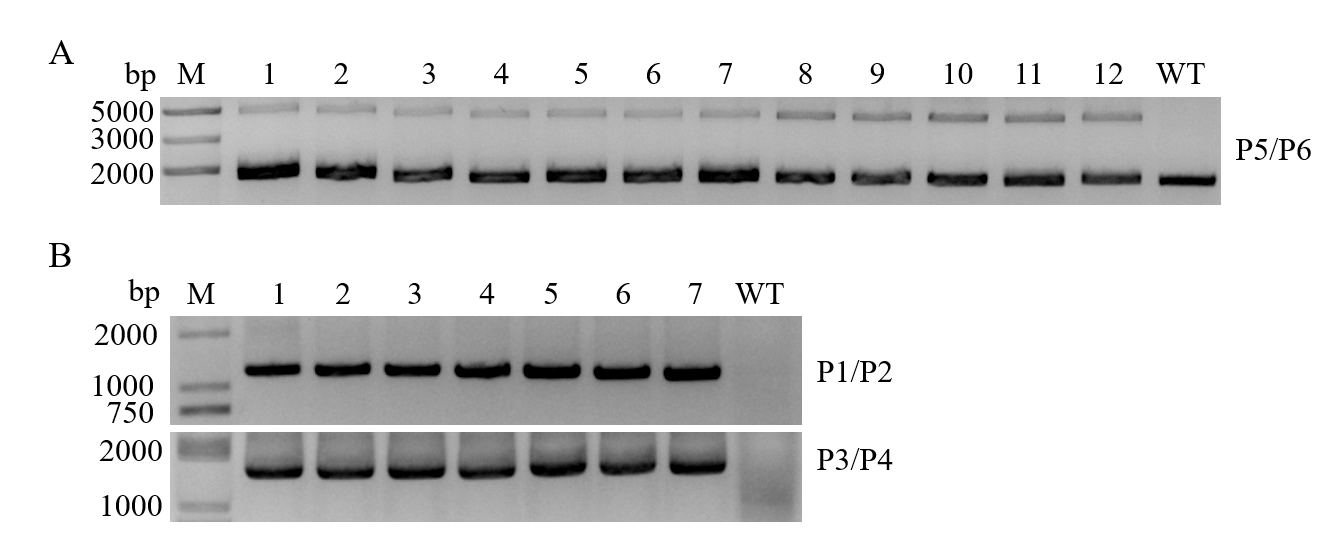


**Figure S3 Genotyping of cell clones and genetically modified pigs by PCR**

A: Genotyping of 12 cell clones by PCR using P5/P6 primers. Lane 1-12 represent different cell clones; WT, wild-type cell lines; M, DL5000; B: Genotyping of genetically modified pigs by PCR using P1/P2 and P3/P4 primers. Lane 1-7 represent 7 individuals; WT, wild-type pig; M, DL2000 Marker.
